# Treatment of Sebaceous Hyperplasia by High-Frequency Focused Ultrasound (HIFU): A Comprehensive Exploration with Clinical Insights

**DOI:** 10.3390/jcm14041305

**Published:** 2025-02-16

**Authors:** Bartosz Woźniak, Natalia Sauer, Anna Pogorzelska-Antkowiak, Piotr Dzięgiel, Jacek Calik

**Affiliations:** 1 Old Town Clinic, 50-136 Wrocław, Poland; 2 Department of Surgical Oncology, Lower Silesian Oncology, Pulmonology and Hematology Center, 53-413 Wrocław, Poland; 3 Department of Clinical Pharmacology, Faculty of Pharmacy, Wroclaw Medical University, Wroclaw Medical University, 50-556 Wrocław, Poland; 4 Department of Clinical Oncology, Wroclaw Medical University, 50-556 Wrocław, Poland; 5 EsteDerm Private Dermatology Clinic, 43-100 Tychy, Poland; 6 Division of Histology and Embryology, Department of Human Morphology and Embryology, Wroclaw Medical University, 50-368 Wrocław, Poland; 7 Department of Human Biology, Faculty of Physiotherapy, Wroclaw University of Health and Sport Sciences, 51-612 Wrocław, Poland

**Keywords:** sebaceous hyperplasia, high-intensity focus ultrasound (HIFU), dermoscopicanalysis, non-invasive skin lesion treatment

## Abstract

**Background:** Sebaceous hyperplasia (SH) is a common benign condition characterized by enlarged sebaceous glands, often requiring treatment for cosmetic or symptomatic reasons. Conventional therapies include laser ablation, electrosurgery, and cryotherapy, but these may be associated with discomfort, scarring, or recurrence. High-Frequency Focused Ultrasound (HIFU) has emerged as a non-invasive alternative. This study evaluates the efficacy, safety, and patient acceptability of HIFU for the treatment of SH. **Methods:** Six patients with a total of 33 SH lesions underwent a single HIFU session at a frequency of 20 MHz. Lesion resolution was assessed clinically and dermatoscopically, while secondary outcomes included patient-reported pain levels and treatment-related side effects. Follow-up evaluations were conducted to determine therapeutic response and tolerability. **Results:** Post-treatment, 87.9% (*n* = 29) of lesions achieved complete resolution, while 12.1% (*n* = 4) demonstrated partial reduction in size. Patients reported minimal discomfort during the procedure, describing it as virtually painless. The side effect profile was favorable, with no significant adverse events recorded. **Conclusions:** HIFU at 20 MHz is an effective and well-tolerated treatment for SH, offering high rates of lesion clearance with minimal discomfort and a low risk of complications. These findings support the role of HIFU as a promising non-invasive alternative to traditional SH treatments, aligning with the trend toward less aggressive dermatological interventions. Further studies with larger cohorts and long-term follow-up are warranted to confirm these results.

## 1. Introduction

Sebaceous hyperplasia, characterized by benign proliferation of sebaceous glands, predominantly manifests on the face, upper neck, and chest of middle-aged to elderly individuals [1]. Sebaceous glands are composed of acini connected to a secretory duct [2]. Sebum production takes place within the sebocyte, where lipids accumulate throughout its life. Eventually, these contents are discharged into the main excretory duct. Clinically, these lesions present as small, yellowish, dome-shaped papules with a distinctive umbilication, often measuring 2–4 mm in diameter. The central umbilication represents the ductal opening, and telangiectasia may be present. These lesions pose a diagnostic challenge as they can be easily mistaken for more serious conditions like basal cell carcinomas [3,4]. Dermoscopically, sebaceous hyperplasia can be identified by specific features. The cumulus sign, an asymmetrical milky-white structure, indicates accumulated sebum due to gland proliferation. The “bonbon toffee sign”, a central umbilication surrounded by the cumulus sign, was observed in a significant proportion of cases, providing an easily recognizable dermatoscopic feature [5]. Crown vessels, fine non-arborizing branching vessels surrounding white or yellow nodules, are also characteristic of sebaceous hyperplasia [6]. The differentiation of sebaceous hyperplasia from other skin lesions, including sebaceous adenoma, sebaceous carcinoma, basal cell carcinoma, and molluscum contagiosum, is crucial and is often aided by dermatoscopy [3].

The pathophysiology of sebaceous hyperplasia is complex and multifactorial. Hormonal changes, particularly those related to androgens, play a significant role [7,8]. During puberty, increased androgen activity leads to the enlargement and activation of sebaceous glands [9]. In contrast, the aging process is marked by a reduction in androgen levels and a consequent decrease in sebocyte regeneration, contributing to the hyperplasia of facial sebaceous glands [10]. Intrinsic factors, including insulin, Thyroid-Stimulating Hormone (TSH), and hydrocortisone, have also been associated with increased proliferation of sebocytes [11]. Additionally, external factors like ultraviolet radiation and immune suppression, particularly with drugs like ciclosporin “A”, play a contributory role in SH proliferation [7,12].

Current treatments for sebaceous hyperplasia focus primarily on cosmetic improvement, encompassing a range of options such as shave excision, isotretinoin therapy, cryotherapy, and various laser treatments [11,13,14,15]. However, these methods often result in recurrence, scarring, or other complications, indicating a need for more effective and enduring therapeutic approaches. In this context, High-Frequency Focused Ultrasound (HIFU) emerges as a novel and promising method [16].

HIFU offers the potential for a less invasive, more targeted treatment, minimizing the drawbacks of traditional approaches. High-Frequency Focused Ultrasound (HIFU) exerts its therapeutic effects through a combination of thermal and mechanical mechanisms. The thermal effect arises from the absorption of ultrasonic energy, leading to rapid heating of tissues at the focal point, with temperatures exceeding 60 °C, causing protein denaturation, coagulative necrosis, and cell death. The mechanical effects are primarily mediated by acoustic cavitation, where the alternating high-pressure (compression) and low-pressure (rarefaction) phases of the ultrasound wave generate microbubbles within the targeted tissue. These microbubbles undergo oscillation and violent collapse (inertial cavitation), producing intense shear forces, shock waves, and localized mechanical disruption of cellular structures, independent of thermal injury. Additionally, acoustic radiation forces generated by the focused ultrasound waves can induce microstreaming and tissue displacement, contributing to vascular occlusion and increased cellular stress. This combination of thermal ablation and mechanical stress results in precise, localized tissue destruction, making HIFU an effective modality for non-invasive treatments across dermatological, oncological, and aesthetic applications [17]. This paper aims to explore the efficacy and safety of HIFU in the treatment of sebaceous hyperplasia, examining its potential to revolutionize the management of this common dermatological condition, thereby improving patient outcomes and quality of life.

## 2. Materials and Methods

Participants diagnosed with sebaceous hyperplasia were recruited and provided informed consent, adhering to the ethical guidelines of the Helsinki Declaration II. In this study, six participants were enrolled, comprising five women and one man, aged 39, 40, 40, 41, 60, and 60 years old. Collectively, these individuals presented with a total of 33 sebaceous hyperplasia lesions. Prior to HIFU treatment, lesions were evaluated using a 20 MHz Dermascan^®^ C ultrasound system and the FotofinderMedicam 1000 (FotoFinder Systems GmbH, Bad Birnbach, Germany) for clinical assessment, ensuring precise characterization of each lesion. Collectively, these individuals presented with a total of 33 sebaceous hyperplasia lesions, providing a diverse sample for the evaluation of the HIFU treatment’s efficacy.

The HIFU treatments were performed with the TOOsonix System ONE-M, operating at 20 MHz [18]. This system features advanced real-time optical monitoring, integrating dermoscopic observation for accurate energy delivery. The transducer chamber was prepared with non-gaseous distilled water, sealed with polyethylene film, and acoustic coupling was achieved with ultrasound gel. We used a 0.8 mm probe, administering HIFU doses of 1 Joule per dose in a contiguous pattern, with each dose lasting 150 ms. A spatial separation of 1 mm between doses ensured comprehensive coverage while preserving the tissue.

The HIFU device, capable of inducing localized heating to approximately 60–65 °C, was expected to cause cell necrosis and denaturate fibrous tissue. This noninvasive approach aimed at efficiently targeting features in the dermis and epidermis. Real-time monitoring via an integrated dermoscopic system displayed on a computer screen facilitated the progress tracking of the treatment. No pre-treatment anesthesia was used, based on prior experience.

## 3. Results

In dermoscopic terms, sebaceous hyperplasia presents as whitish-yellowish nodular structures, typically encircled by a crown-like arrangement of blood vessels. These vessels notably do not traverse the lesion’s central part.

**Figure 1:**
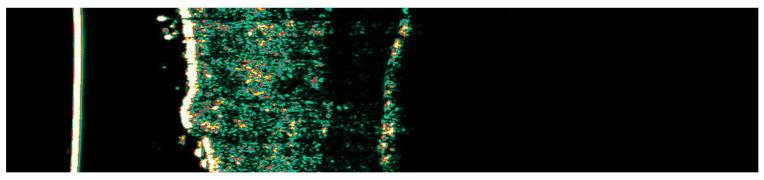
Ultrasonographic image of sebaceous hyperplasia.

Figure 1 presents a pre-treatment ultrasound measurement of SH in one of our patients, performed using the Dermascan^®^ C ultrasound system. SH typically appears as a hyperechoic structure within the upper dermis on ultrasound imaging. Accurately determining lesion depth is a crucial aspect of pre-treatment assessment, as individual variations exist. While most cases of SH are superficially localized (0.3–1.0 mm), some lesions may extend deeper, depending on their anatomical location. In our patient cohort, SH lesions were found at depths ranging from 0.3 mm to 0.7 mm. Based on these measurements, a 0.8 mm probe was used for all procedures to ensure optimal targeting and treatment efficacy.

Post-procedure, an inflammatory reaction is observed, characterized by pink discoloration and a pattern of reticular and linear branching vessels. Dermoscopic follow-up often reveals white amorphous areas and white lines indicative of fibrosis, arranged perpendicular to the skin’s surface. Over time, a transient depression may form at the treatment site, which eventually undergoes a regenerative process, restoring the skin’s original contour.

**Figure 2:**
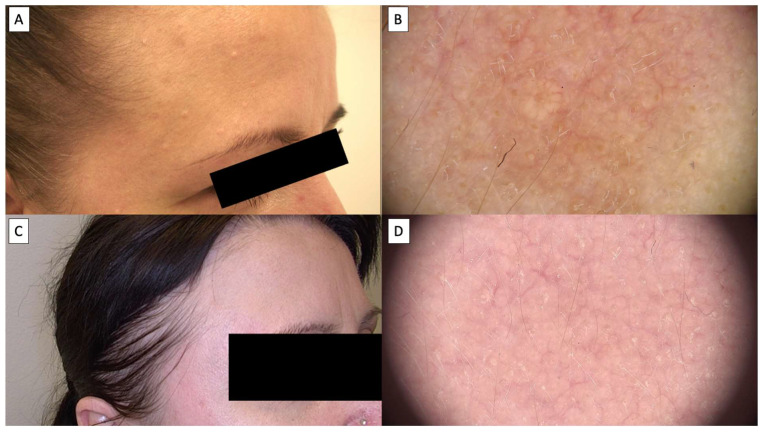
(**A**) Macroscopic view of sebaceous hyperplasia before treatment; (**B**) dermatoscopic view sebaceous hyperplasia before treatment; (**C**) macroscopic view of sebaceous hyperplasia 3 months after HIFU treatment; (**D**) dermatoscopic view of sebaceous hyperplasia 3 months after HIFU treatment.

**Figure 3:**
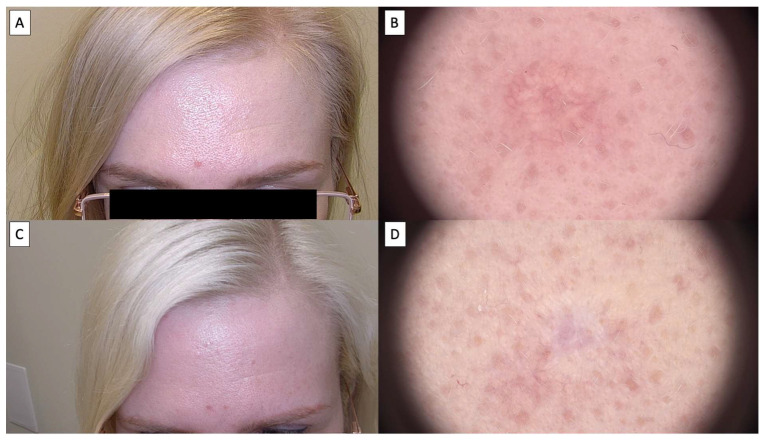
(**A**) Macroscopic view of sebaceous hyperplasia before treatment; (**B**) dermatoscopic view of sebaceous hyperplasia before treatment; (**C**) macroscopic view of sebaceous hyperplasia 3 months after HIFU treatment; (**D**) dermatoscopic view of sebaceous hyperplasia 3 months after HIFU treatment.

**Figure 4:**
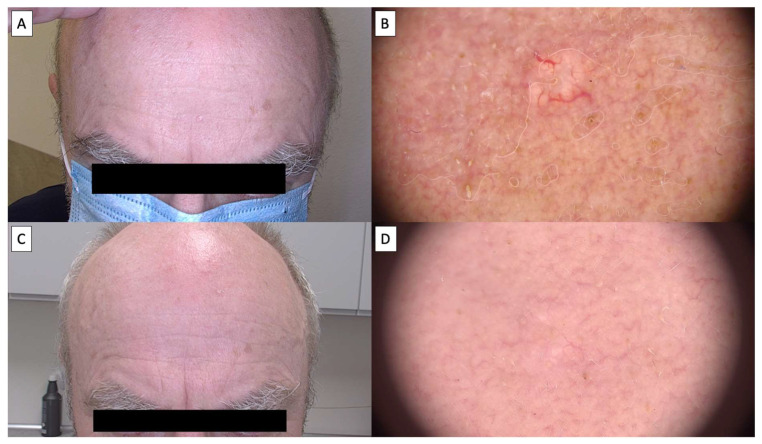
(**A**) Macroscopic viewof sebaceous hyperplasia before treatment; (**B**) dermatoscopic view of sebaceous hyperplasia before treatment; (**C**) macroscopic view of sebaceous hyperplasia 3 months after HIFU treatment; (**D**)dermatoscopic view of sebaceous hyperplasia 3 months after HIFU treatment.

**Figure 5:**
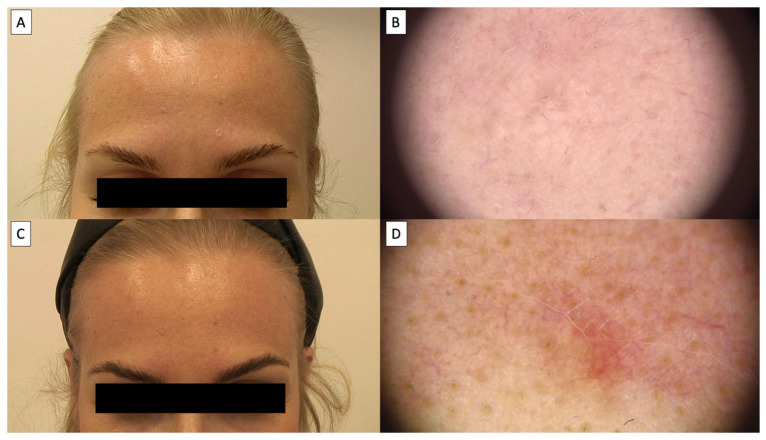
(**A**) Macroscopic view of sebaceous hyperplasia before treatment; (**B**) dermatoscopic view of sebaceous hyperplasia before treatment; (**C**) macroscopic view of sebaceous hyperplasia 3 months after HIFU treatment; (**D**) dermatoscopic view of sebaceous hyperplasia 3 months after HIFU treatment with visible telenagiectasias.

Dermoscopic assessment after HIFU therapy showed that the majority of patients achieved complete resolution of sebaceous hyperplasia, as depicted in Figure 2, Figure 3, Figure 4 and Figure 5. Notably, the characteristic dermoscopic appearance of these lesions as whitish-yellowish nodular structures encircled by a distinct vascular pattern known as the “crown sign” was observed to resolve following treatment. Figure 2 illustrates an ideal healing process with an excellent post-procedural outcome, both macroscopically (C) and dermatoscopically (D). While most cases resulted in total lesion clearance without any adverse effects, a subset of patients developed minor side effects, which were meticulously documented in Table 1. Figure 3 shows an SH lesion previously treated with a CO_2_ laser in a female patient, which recurred after one month. In our case, the lesion was completely removed both macroscopically (C) and dermatoscopically (D). However, white lines indicating scarring are visible. Other side effects included the formation of small telangiectasias, indicated by Figure 5, as well as minor scarring and hyperpigmentation, all of which were localized and limited to the area of treatment. Figure 4 shows the complete macroscopic removal of the lesion (C). However, under dermatoscope (D), a small remnant of a large arborizing vessel is visible. The lesion requires further follow-up to exclude potential recurrence in the future. This also highlights the role of dermoscopy in monitoring treatment outcomes and detecting lesions with a higher risk of recurrence.

Patients universally reported the treatment to be virtually painless, likening the sensation to that of a slight needle prick. This patient feedback highlights the tolerability and minimal discomfort associated with the HIFU procedure. Over time, dermoscopic follow-up demonstrated a transient tissue depression at the treatment sites, which progressively regenerated, reinstating the skin’s original contour, as illustrated in Figure 2, Figure 3, Figure 4 and Figure 5. The images showcase the typical inflammatory response characterized by pink discoloration and a pattern of reticular and linear branching vessels, which transition to white fibrotic lines and amorphous areas perpendicular to the skin surface, indicating the onset of the healing process.

In the study involving six patients with a total of 33 sebaceous hyperplasia lesions, the results demonstrated the effectiveness of HIFU treatment. Approximately 87.9% (*n* = 29) of the lesions achieved a complete response (CR), signifying complete clearance, while approximately 12.1% (*n* =4) exhibited a partial response (PR) with a partial reduction in size or severity. None of the lesions remained in a stable condition (SC) or showed progressive condition (PC), underscoring the favorable outcomes and safety profile of HIFU therapy for sebaceous hyperplasia in this patient cohort.

## 4. Discussion

The present study’s exploration into the utility of High-Frequency Focused Ultrasound (HIFU) for treating sebaceous hyperplasia (SH) marks a significant contribution to dermatological therapeutics. Our findings, showcasing HIFU’s efficacy in lesion reduction with minimal adverse effects, resonate with the emerging narrative in dermatological research that advocates for less invasive treatment modalities. This alignment is particularly evident when juxtaposed with the existing literature on CO_2_ laser treatments for SH, which, while effective, often come with a higher incidence of complications such as scarring and pigmentation changes [19].

The efficacy of High-Frequency Focused Ultrasound (HIFU) in treating sebaceous hyperplasia, as demonstrated in our study, aligns with emerging trends in dermatological therapy, particularly in non-invasive skin treatments. HIFU’s utility, as explored in diverse contexts ranging from benign vascular tumors to facial skin rejuvenation, highlights its versatility [20,21,22,23,24]. Even larger nodules, such as neurofibromas in the course of neurofibromatosis type I, can be effectively treated using this method [25]. There are also preliminary studies demonstrating the efficacy of HIFU in treating malignant tumors, such as basal cell carcinoma, as well as premalignant conditions like actinic keratosis [26,27]. Given its ability to precisely target superficial and dermal structures, HIFU may also hold potential for treatingtrichodiscomas in Birt−Hogg−Dubé syndrome, angiofibromas in tuberous sclerosis, as well as cosmetic concerns such as milia (excluding the periorbital region).These applications further underscore the growing role of HIFU in dermatology as a non-invasive and selective therapeutic approach.

Moreover, the advancements in diagnostic accuracy for sebaceous hyperplasia, as indicated by Lenoir et al., underscore the importance of precise therapeutic interventions [28]. HIFU, in this regard, offers a targeted approach, minimizing the risk of overtreatment—a common challenge in managing skin lesions [29,30]. Our results emphasize the minimal side effects and high patient satisfaction associated with HIFU. This aspect is particularly significant given the chronic and often recurrent nature of sebaceous hyperplasia, where patient quality of life is a key treatment outcome [11]. In the event that not all white-yellow deposits within sebaceous hyperplasia (SH) lesions are fully eliminated, there is a possibility of recurrence, potentially necessitating another round of treatment [19]. Thus, the precision afforded by 20 MHz HIFU is crucial; it allows for the application of high-power ultrasound energy to very small focal targets in the dermis, thereby ensuring precise confinement of thermal lesions and reducing the likelihood of incomplete treatment and subsequent recurrence [31,32]. In particular, the use of HIFU for the sebaceous gland removal was found to have impressive outcomes and provide minimal damage to surrounding tissues.

Further observations and studies are needed to better understand the healing process following HIFU treatment. It is known that the healing process may depend on the type of lesion itself—for example, sebaceous hyperplasia statistically heals faster than seborrheic keratosis or sebaceous nevus [33]. Healing time may also vary, depending on the lesion’s location within the face. Lesions on bony prominences (e.g., the forehead or zygomatic bone) may take longer to heal and result in a less favorable cosmetic outcome compared to those in concave areas (e.g., the temple). So far, we have estimated that the average healing time for sebaceous hyperplasia (SH) is approximately three weeks. The final outcome may also be influenced by the baseline skin condition (such as the degree of photodamage) and post-treatment skincare. As with lasers and other physical methods for removing skin lesions, photoprotection plays a crucial role in preventing hyperpigmentation and telangiectasias, which can be exacerbated by UV radiation.

The revised discussion emphasizes the patient-centric approach of the study, highlighting the minimal invasiveness and patient satisfaction with HIFU treatment for sebaceous hyperplasia (SH). It aligns with current trends in dermatology that prioritize patient quality of life and clinical efficacy. This study reinforces HIFU as a non-invasive, viable alternative for SH treatment, encouraging further research into its long-term efficacy and comparison with other methods. The study opens avenues for exploring HIFU’s broader dermatological applications, suggesting a significant impact on clinical practices by offering a safe, effective, patient-friendly treatment option.

## 5. Conclusions

While we acknowledge the limitation of a small patient cohort and the associated challenges in interpreting the results, our findings demonstrate the promising potential of HIFU for the treatment of sebaceous gland hyperplasia. This study provides a foundation for future controlled trials with larger patient groups, which will be essential for confirming its long-term efficacy and safety. Additionally, comparative studies with CO_2_ laser and chemical peels—currently the most common treatment modalities for SH—will help establish HIFU’s relative effectiveness and clinical applicability. Our results indicate that 20 MHz HIFU is an effective, non-invasive alternative for SH treatment, offering high lesion clearance rates with minimal discomfort and side effects. Given its precise targeting capabilities and favorable safety profile, HIFU has the potential to become a valuable addition to the dermatological treatment landscape. Future studies should focus on optimizing treatment parameters and evaluating long-term outcomes to further define its role in clinical practice.

## Figures and Tables

**Table 1:** Patient outcomes following HIFU treatment for sebaceous hyperplasia. “CR” indicates complete response (total lesion clearance), “PR” stands for partial response (partial reduction in lesion size or severity), “SC” means stable condition (no significant change in the lesion), and “PC” denotes progressive condition (lesion growth or worsening). Side effects experienced post-treatment are also noted. Each row details individual lesion outcomes by patient number, gender, lesion location, and follow-up results.

Subject	Gender	Lesion Number	Location	Outcome at the Final Follow-up				Side Effects
				CR	PR	SC	PC	
1	F	1	cheek	yes				none
		2	temple	yes				small teleangiectasias
		3	temple	yes				none
		4	forehead	yes				none
		5	forehead	yes				none
		6	forehead	yes				none
		7	forehead	yes				none
		8	forehead	yes				none
		9	forehead	yes				none
		10	forehead		yes (75%)			none
		11	forehead	yes				none
2	F	1	forehead	yes				small scaring
3	F	1	temple	yes				none
		2	forehead	yes				small teleangiectasias
		3 (double)	forehead	yes				none
		4	forehead	yes				none
		5	forehead	yes				none
		6	forehead	yes				small hyperpigemantation
4	F	1	cheek	yes				small teleangiectasias
		2	temple	yes				small hyperpigemantation
5	M	1	forehead	yes				none
6	F	1	cheek		yes (reduction in thickness by 50%)			none
		2 (double)	temple	yes				small teleangiectasias
		3	temple	yes				erythema
		1 (triple)	forehead	yes				none
		2	forehead		yes (reduction in thickness by 50%)			none
		3	forehead	yes				none
		4 (double)	forehead	yes (1)	yes (75%)			none

## Data Availability

The data are contained within the article; further inquiries can be directed to the corresponding author.
